# Impact of Caffeine Intake on 800-m Running Performance and Sleep Quality in Trained Runners

**DOI:** 10.3390/nu11092040

**Published:** 2019-09-01

**Authors:** Domingo Jesús Ramos-Campo, Andrés Pérez, Vicente Ávila-Gandía, Silvia Pérez-Piñero, Jacobo Ángel Rubio-Arias

**Affiliations:** 1 Faculty of Sports, UCAM, Catholic University San Antonio, 30107 Murcia, Spain; 2 High Performance Research Center (CIARD), UCAM, Catholic University San Antonio, 30107 Murcia, Spain; 3 Department of Exercise Physiology, Catholic University San Antonio, 30107 Murcia, Spain

**Keywords:** actigraphy, athletic, coffee, ergogenic aid, supplement

## Abstract

Background: Caffeine ingestion improves athletic performance, but impairs sleep quality. We aimed to analyze the effect of caffeine intake on 800-m running performance, sleep quality (SQ), and nocturnal cardiac autonomic activity (CAA) in trained runners. Methods: Fifteen male middle-distance runners participated in the study (aged 23.7 ± 8.2 years). In a randomized and comparative crossover study design, the athletes ingested a placebo (PL) or caffeine supplement (CAF; 6 mg∙kg^−1^) one hour before an 800-m running time-trial test in the evening. During the night, CAA and SQ were assessed using actigraphy and a sleep questionnaire. A second 800-m running test was performed 24 h after the first. Time, heart rate, rating of perceived exertion, and blood lactate concentration were analyzed for each running test. Results: No significant differences in CAA and performance variables were found between the two conditions. However, CAF impaired sleep efficiency (*p* = 0.003), actual wake time (*p* = 0.001), and the number of awakenings (*p* = 0.005), as measured by actigraphy. Also, CAF impaired the questionnaire variables of SQ (*p* = 0.005), calm sleep (*p* = 0.005), ease of falling asleep (*p* = 0.003), and feeling refreshed after waking (*p* = 0.006). Conclusion: The supplementation with caffeine (6 mg∙kg^−1^) did not improve the 800-m running performance, but did impair the SQ of trained runners.

## 1. Introduction

Scientists and coaches are continually looking for techniques to develop more effective and efficient methods to improve exercise performance [1]. One of the popular methods commonly used by athletes to maximize their physical performance is the intake of legal ergogenic aids [2]. In this way, caffeine is frequently used in sport as an ergogenic aid to improve athletic performance and endurance [3]. In fact, it has been reported that 74% of elite athletes may use caffeine as an ergogenic aid prior to or during a competition [4]. Caffeine is a xanthine alkaloid that increases central nervous activity by the blockade of central and peripheral adenosine receptors [5]. This stimulant action produces a greater recruitment of motor units [6], improves the Na^+^–K^+^ pump response [7], and increases the rate of calcium release from the sarcoplasmic reticulum [8] and the mobilization of free fatty acids [9]. Also, caffeine enhances adrenaline secretion [10] and reduces ratings of perceived exertion [11]. Therefore, caffeine is administered in order to improve sport performance.

Previous studies that analyzed the effect of caffeine ingestion on runners have shown improvements in running performance compared to placebo [12,13]. It had previously been reported that compared to placebo, the intake of 4.5 mg∙kg^−1^ of caffeine increased exercise distance by 2–3 km when running at 85% maximum oxygen uptake until exhaustion [10]. Regarding middle-distance races, compared to placebo, 1500-m [13] or one-mile [14] running performances are improved by 1.3–1.9% after 150–200 mg and 3 mg∙kg^−1^ of caffeine intake, respectively. However, another study found similar 800-m running performance in amateur runners after placebo or 5.5 mg∙kg^−1^ of caffeine administration [15]. Thus, there is conflicting evidence in relation to the effectiveness of caffeine as an ergogenic aid to improve middle-distance race performance in athletes.

On the other hand, caffeine intake can impair sleep [16], which is considered the most important method for recovery from daily load [17]. Sleep assists in the recovery of the nervous and metabolic cost imposed by the waking state [18]. However, caffeine typically prolongs sleep latency, reduces total sleep time and sleep efficiency, and worsens perceived sleep quality (SQ) [16], particularly if it is administered close to bedtime. Moreover, vigorous-intensity exercise completed close to bedtime increases the latency time and impairs SQ [19]. Therefore, the use of caffeine as an ergogenic aid in a competition performed close to bedtime may decrease SQ and the recovery process, which may decrease athlete performance on the following day. There are some sports modalities, such as athletics, where the athlete needs to perform in qualification races over consecutive days. Some of these races are performed at the end of the evening, and the rest time between the first race (e.g., a semi-final) and the following one (e.g., the final) may be very short. For example, during the Athletics World Championships of 2019, the qualification and the semi-final race of the 800-m event were separated by 24 h. Thus, the administration of caffeine before a qualification race performed in the evening may affect the recovery process and performance in the races on the following day due to sleeping problems. However, there are no studies that have analyzed the effect of caffeine administration to aid performance in a race close to bedtime on SQ and on the running performance the following day.

Therefore, the aim of the present study was to analyze the effect of caffeine intake one hour (19:00 h) before an 800-m race (20:00 h) on actigraphic SQ, subjective SQ, and nocturnal cardiac autonomic activity (CAA), and on the 800-m performance performed 24 h later in trained middle-distance athletes. We hypothesized that the pre-exercise ingestion of 6 mg of caffeine per kg of an athletes’s body mass would impair SQ through subjective and actigraphic impairment, but it would not affect the race performance on the following day.

## 2. Methods

### 2.1. Design

A randomized and comparative crossover study was conducted to test the effects of caffeine intake or placebo before an 800-m running time trial on actigraphic SQ, the subjective quality of sleep, nocturnal autonomous cardiac activity, countermovement jump (CMJ), and the 800-m performance of athletes at international and national levels. Athletes reported to their usual official athletics track four times over two consecutive weeks. The testing sessions were developed during two consecutive Friday and Saturday evenings in March. Two weeks before the study, the athletes had finished their winter season, performing in the National Indoor Championships. Therefore, the study was developed in a general period training phase.

Upon arrival at the athletics track, runners were given a caffeine or a placebo supplement—placebo (PL) or caffeine (CAF) in randomized order—in experimental Sessions 1 and 3, while no supplements were taken in the experimental Sessions 2 and 4. Forty-five minutes (min) after the intake of the supplements in Sessions 1 and 3, or 45 min after the runners arrived at the athletics track, the participants started the testing session.

An 800-m running time-trial test was performed in each testing session. Performance (time, CMJ height), physiological (peak and mean heart rate and blood lactate concentration), and subjective (rating of perceived exertion) variables were collected during the testing session. The sessions were carried out at 20:00 h and under similar environmental conditions (20–22 °C). In addition, we used actigraphy to monitor the night after PL or CAF ingestion to assess SQ and a sleep questionnaire and to analyze the autonomic modulation.

### 2.2. Participants

Fifteen male runners in mid-level events participated in the study (age: 23.7 ± 8.2 years; height: 177.4 ± 9.0 cm; weight: 64.6 ± 9.8 kg). Runners performed 9.0 ± 1.8 h per week of training and had at least six years of middle-distance training experience. They were of national and international standard at the 800-m level and their best time at that distance ranged between 1:46.72–2:04.10. Eleven of the runners were of Caucasian race, two were from North Africa (Maghreb race), one was from South America (Latino race), and another was from Central Africa (Black race). All the subjects gave their signed and informed consent, and the study was approved (CE031909) by the Ethics Committee in Institutional Sciences of the University and was in accordance with the Declaration of Helsinki. The subjects were asked to maintain their usual diet and hydration status and not to ingest caffeine or alcohol at least 24 h before each test session or to carry out exhaustive training in the 48 h prior the first and third testing sessions.

### 2.3. Procedures

Athletes ingested a placebo (sucrose) or caffeine supplement (6 mg∙kg^−1^) in capsules of the same size, color, and smell in a typical double-blind trial, with a 50% chance of ingesting the actual active or placebo substance, avoiding any effects of session or time on the results. The blinding efficacy was checked after the participants had finished their participation. In addition, participants were issued with nutritional guidelines to ensure that they followed a similar diet in the 48 h before each condition session. This diet was the same that runners usually used during competition. The last meal was eaten by runners 3 h before the test. Furthermore, 24 h before each experimental session, caffeine ingestion was restricted. In addition, a caffeine consumption questionnaire [20] was administered to the runners, which showed that all the runners were daily consumers of caffeine (between 250–572 mg of caffeine∙day^−1^) according to classification proposed elsewhere [21]. Also, all the runners were used to ingesting caffeine (6 mg∙kg^−1^) as an ergogenic aid prior to competition.

### 2.4. Testing Session

During the first visit, body composition was evaluated using a bioimpedance segmental analyzer (Tanita BC-601, Tanita Corp, Tokyo, Japan) following previous recommendations [22]. In addition, 45 min after supplement ingestion, participants performed their traditional competitive warm-up of 15 min duration, including running at low intensity, joint mobility, dynamic stretching, and progressive running sets. After warm-up, a CMJ test was carried out. Two minutes later (~60 min after supplement ingestion), an 800-m time-trial test was performed. Finally, 2 min after the end of the running test, a blood lactate concentration analysis and another CMJ test were carried out. The mean and peak heart rates (Polar RS800, Polar Electro Oy, Kempele, Finland) were recorded during the 800-m running time trial. In addition, ratings of perceived exertion (RPE) were determined using the 10-point Borg scale [23] following the 800-m time trial. The 800-m times were recorded using a Geonaute chronometer Onstart 710 (Decathlon, Villeneuve-d’Ascq, France) by two of the researchers, and the mean of these values was used for analysis. Capillary blood samples (5 μL) were collected by finger prick 2 min after the end of the running test and analyzed for blood lactate concentration ([La–]) using a Lactate Pro analyzer (Lactate Pro, Arkay, Inc., Kyoto, Japan). Countermovement jump heights were performed using a contact platform (Ergotester, Globus, Codogne, Italy). The participants executed two submaximal trials to ensure proper execution of the jumps with 1-min rest between trials. The CMJ height was measured before warm-up and prior to the 800-m time trial, and performed at the center of the platform with the feet placed shoulder-width apart in the standing position. Participants were asked to jump as high as possible with a rapid self-selected countermovement. The depth of the countermovement was self-selected, and participants were asked to try to land close to the take-off point. Each individual’s best performed was used for data analysis. The same testing procedure was applied in each testing session.

### 2.5. Actigraphic Quality of Sleep, Subjective Quality of Sleep, and Autonomous Nocturnal Cardiac Activity

Between the end of testing session and the time to go to bed, the athletes had to do their normal life and record any activity in a diary. Participants were instructed to measure actigraphic sleep quality and nocturnal cardiac autonomic activity (Heart Rate Variability-HRV) during sleep after each day with a training session day. Actigraphic sleep quality was recorded using an actiwatch activity monitoring system (Cambridge Neurotechnology, Cambridge, UK), which measures activity by means of a piezoelectric accelerometer. The movement of the non-dominant wrist of each participant was monitored. A low actigraphic sensitivity threshold (80 counts per epoch) was selected, and the data recorded by the actigraph were analyzed with Actiwatch Sleep Analysis Software. Each subject received a sleep diary to record bedtime, wake-up time, hours napping, hours without wearing the actigraph, and the number of nocturnal awakenings. Data analysis started with the onset of nocturnal rest (bedtime) and ended with the onset of daytime activity (wake time). The following sleep parameters were measured: (I) sleep efficiency (%): percentage of time spent asleep; (II) time in bed (min); (III) actual sleep time (min); (IV) actual wake time (min); (V) number of awakenings; (VI) average time of each awakening(min); and (VII) latency.

Together with the actigraph, during the night, each subject wore an H7 strap Heart monitor (Polar Electro, Kempele, Finland) to evaluate HRV. Variables of cardiac autonomic activity were analyzed for the 4-h period of sleep starting 30 min after the reported bedtime [20]. The R–R series were analyzed using Kubios HRV software (version 2.0, Biosignal Analysis and Medical Imaging Group, University of Kuopio, Finland). The following HRV variables were assessed: (I) low-frequency (LF) band / high-frequency (HF) band ratio; (II) total power (TP); (III) percentage of differences between adjacent normal R–R intervals more than 50 ms (pNN50); (IV) square root of the mean of the sum of the squared differences between adjacent normal R–R intervals (RMSSD); (V) standard deviation of all normal N–N intervals (SDNN); (VI) mean heart rate; and (VII) mean R–R intervals.

Participants were also instructed to evaluate their subjective sleep quality in the morning after awakening using the Karolinska Sleep Diary [24], which analyzes the following questions: (I) sleep quality (very well [5] to very poorly [1]); (II) calm sleep (very calm [5] to very restless [1]); (III) ease of falling asleep (very easy [5] to very difficult [1]); (IV) amount of dreaming (much [3] to none [1]); (V) ease of waking up (very easy [5] to very difficult [1]); (VI) feeling refreshed after awakening (completely [3] to not at all [1]); (VII) slept throughout the time allotted (yes [5] to woke up much too early [1]).

### 2.6. Statistical Analysis

Statistical analysis of data was performed with SPSS 21.0 software (SPSS 21.0, Chicago, IL, USA) in a Windows environment. Descriptive data are presented as mean ± SD and range. For inferential analysis, a Shapiro–Wilk W-test was performed to establish the normality of the sampling distribution, and Mauchly’s W-test analyzed the sphericity between measurements. In addition, analysis of variance for repeated measures (ANOVA) was calculated (general linear model) to analyze the effects of caffeine intake on performance over 800 m, and a paired sample T-test or the nonparametric equivalent (Wilcoxon test) was used to compare the effect of caffeine on heart rate variability and SQ. Effect size (ES) was calculated using partial eta-squared (η^2^p) for variance analysis and Cohen’s d to indicate the standardized difference between two means. Threshold values for ES were ≥0.1 (small), ≥0.3 (moderate), ≥1.2 (large), and ≥2.0 (very large) [25]. The level of significance was set at *p* ≤ 0.05.

## 3. Results

**Figure 1:**
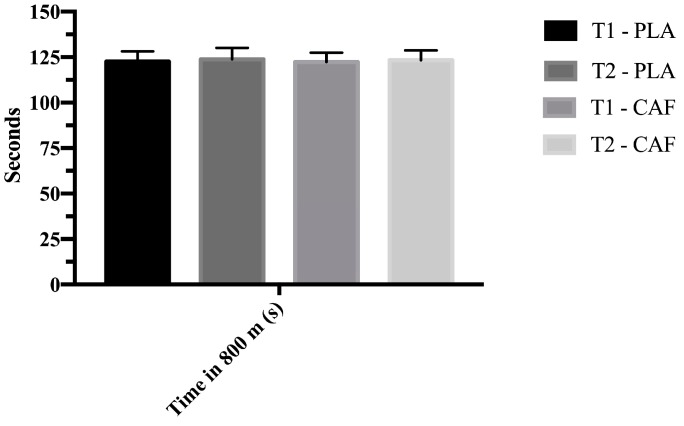
Time in 800 m (s). T1: First test 1; T2: second test; PLA: Placebo; CAF: Caffeine.

Table 1 presents the summary statistics for the changes in performance under each of the measured conditions (placebo and caffeine). No significant effects were found in performance (Figure 1).

Significant effects were observed in the variable CMJ (F = 4.564; *p* = 0.008) with a large effect size (η^2^p = 0.28); the pair comparison showed a significant difference between the CMJ results (Δ) on days 1 and 2 when participants took caffeine (mean differences = −6.51, t = −3.14, *p* = 0.020). However, no significant effects were found in in any other variable.

**Figure 2:**
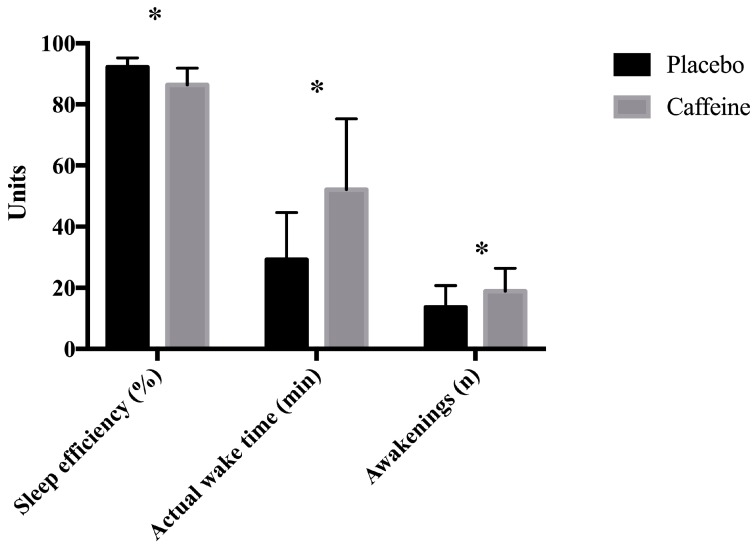
Sleep quality results measured by actigraphy * Significant differences between placebo and caffeine (*p* < 0.05).

Concerning the SQ results, actigraphic analysis showed significant differences between conditions (placebo versus caffeine) in sleep efficiency (*p* = 0.003; ES = 0.71), actual wake time (*p* = 0.001; ES = −1.18), and number of awakenings (*p* = 0.005; ES = −0.96) (Figure 2 and Table 2).

In addition, the Karolinska sleep questionnaire showed significant differences between conditions, favoring placebo in SQ (*p* = 0.005; ES = 1.11), calm sleep (*p* = 0.005; ES = 1.11), ease of falling asleep (*p* = 0.003; ES = 1.38), and feeling refreshed after waking (*p* = 0.006; ES = 1.11) (Table 2).

Table 3 shows the summary statistics for heart rate variability during the night. No significant differences were observed between caffeine and placebo.

## 4. Discussion

To our knowledge, this is the first study to investigate the effects of caffeine intake 1 h (19:00) before an 800-m running time trial (20:00) on actigraphic SQ, subjective SQ, and nocturnal CAA, and on the 800-m performance 24 h later, in trained middle-distance athletes. We found that the ingestion of 6 mg∙kg^−1^ of caffeine did not improve the 800-m running performance. In addition, caffeine intake did not modify the 800-m running performance one day after the first 800-m running test. However, regarding SQ, athletes reported significantly worse subjective SQ, calm sleep, ease of falling asleep, and feeling refreshed after waking after CAF ingestion in comparison to PL. In addition, caffeine ingestion impaired the sleep quantity and quality as measured by actigraphy (reducing sleep efficiency, increasing the number of awakenings, and increasing the actual wake time) in 800-m athletes, but did not affect the autonomic nervous system during the night.

Caffeine is a supplement with good-to-strong evidence of achieving benefits in athletic performance when used in specific scenarios across endurance-based situations and in short-term, supramaximal, and/or repeated sprint tasks [26]. However, our findings revealed no significant differences in 800-m times when caffeine ingestion and placebo were compared. These results are in accordance with a study by Marques et al. [15], who found no performance differences between placebo and caffeine conditions in 800-m time-trial running performance in overnight-fasting runners. Furthermore, recent research has shown no positive effect of 5 mg∙kg^−1^ intake on anaerobic capacity in recreationally active men. Anaerobic capacity is a key factor in performance in middle-distance sports (e.g., 800 m) [27]. In contrast, there are several studies that have found improvements with the use of caffeine as an ergogenic aid in tests of similar metabolic demands [14,28]. These controversial findings could be due to the characteristics of the subjects, their daily caffeine intake, and their experience in the use of caffeine as an ergogenic aid: previous studies have reported that the ergogenic effect of caffeine in habitual caffeine consumers is diminished [29,30]. In addition, several studies have found significant differences when intake and testing is carried out in the morning versus the evening, showing benefits when the protocol was carried out in the morning, and not when it was carried out in the evening [31,32]. Therefore, this must be considered in our study, because the experimental protocol was performed in the evening, which could diminish the potential effects of caffeine. Finally, the genetic predisposition of athletes has been shown to have a great influence on the responses to the intake of this ergogenic aid [33]. Some genetic polymorphisms affect the speed of metabolism of caffeine (CYP1A2) and the excitability of the nervous system (ADORA2A) [33], and this could affect the results obtained in the present study. Therefore, future studies would assess a genetic test to analyze how these polymorphisms affect 800-m running performance. Finally, regarding RPE, our results showed no significant differences between PL and CAF conditions. These findings are in accordance with the above-mentioned 800-m running study [15]. Moreover, our results agree with a previous meta-analysis that concluded that the intake of caffeine produces a significant reduction in RPE during exercise, but does not produce any change at the end of exhausting exercise [34].

Good sleep is vital in the regulation of hormone secretion and in the restoration of metabolic processes in athletes [35]. However, some factors can impair SQ in athletes before a competition: about 66% of athletes report that they often experience worse sleep than usual on the night(s) before a competition [36] for various reasons, including noise, light, anxiety, and nervousness [37]. Moreover, previous studies report that performing intense exercise close to bedtime impairs SQ [19]. In addition, caffeine ingestion may have adverse effects on SQ [3,16]. Interestingly, the current study, using trained male athletes, found that a 6 mg∙kg^−1^ dose of caffeine taken 1 h (19:00) before an 800-m race (20:00) impairs SQ, with lower sleep efficiency and greater actual wake time and number of awakenings. These findings can be due to caffeine promoting wakefulness by antagonizing adenosine A1 and A2A receptors in the brain [38]. These adenosine agonist receptors play a role in arousal and promoting sleep. In addition, 6-sulphatoxymelatonin excretion plays an essential role in in the biological regulation of circadian rhythms, including sleep, and previous studies have reported that caffeine interferes with sleep quantity and quality by the reduction of this substance [39]. Therefore, these physiological responses can explain the SQ results obtained in the present study. Notably, although poor sleep was reported after CAF ingestion, no influence on performance was found. This finding is in accordance with previous studies that reported that disturbed sleep had no influence on sporting performance in competitions [36,40]. Some possible reasons to explain this unchanged exercise performance following a night of poor-quality sleep are that metabolic pathways, rating of perceived exertion, and physiological responses remain largely unaltered [37]. The performance, physiological, and perceptual results of the present study agree with this finding.

Several studies have analyzed the relationship between HRV and caffeine ingestion [41,42], reporting that caffeine seems to produce predominantly a parasympathetic rather than a sympathetic cardiac influence [43]: some studies report that the acute ingestion of caffeine enhances parasympathetic activity [44], and tends to decrease the LF/HF ratio under resting conditions [43], or increase this variable during sleep after caffeine administration [45]. However, other studies found no changes in HRV at rest [46] comparison to placebo. These findings are in accordance with the results of the present study, where no significant differences were observed in HRV variables during sleep after placebo or caffeine ingestion. One possible reason for these findings can be related to the daily caffeine consumption of the participants. Previous studies have reported that the response of the autonomic nervous system to caffeine intake is diminished in habitual caffeine consumers [47]. Therefore, the lack of effect on HRV in the athletes in the present study could be related to the rapid tachyphylaxis of caffeine, as reported [47]. In addition, the effect of caffeine on HRV seems to be time-dependent, resulting in an enhancement of the activity of autonomic nervous system 2.5 h after caffeine ingestion [46]. Thus, in our study the participants ingested the caffeine ~3.5–4.5 h before going to sleep, which could be related to the lack of difference in HRV variables during sleep between the conditions (placebo versus caffeine).

From an application perspective, athletic coaches of middle-distance runners should keep in mind that if the championship has races on consecutive days, the administration of 6 mg∙kg^−1^ of caffeine does not improve the 800-m running performance, but can impair sleep quantity and the quality of trained runners who are habitual caffeine consumers.

The main limitation of the present study was that the number of athletes that took part in the study was limited. In addition, our results cannot be generalized to other subjects who ingest lower amounts of caffeine per day (i.e., light caffeine consumers); neither can our findings can be generalized to other athletes’ modalities (e.g., long distance) or gender (female athletes). Finally, the results of plasma caffeine concentration were not determined. On the other hand, the main strength of the present study is the level of the athletes who participated and the practical application of the results to the real athletic field. Further research into the influence of caffeine supplementation on running performance and recovery processes (e.g., sleep, using electroencephalography) would be necessary. Moreover, it would be interesting to increase the number of subjects in a future study also comparing subjects with regular and non-regular intake of caffeine.

## 5. Conclusions

In comparison to placebo, the ingestion of 6 mg∙kg^−1^ of caffeine did not improve the 800-m running performance in daily consumers of caffeine trained athletes, and did not modify the performance of a subsequent 800-m running test performed one day after the first. However, caffeine impaired the subjective and actigraphic sleep quantity and quality, but did not affect the autonomic nervous system during the night after the participants had performed the first 800-m running test.

## Figures and Tables

**Table 1:** Results of 800-m running time trial test variables.

	Placebo				Caffeine				ANOVA		
	Test 1		Test 2		Test 1		Test 2				
	mean	SD	mean	SD	mean	SD	mean	SD	F	p	η^2^p
Time in 800 m (s)	122.6	5.6	123.8	6.2	122.3	5.1	123.3	5.4	2.317	0.12	0.15
RPE (A.U)	8.4	1.1	8.2	1.0	8.3	0.9	8.1	0.9	0.142	0.934	0.01
mean HR in 800 m (bpm)	170.4	9.8	171.4	10.1	172.7	10.6	173.2	9.2	0.625	0.525	0.06
peak HR in 800 m (bpm)	185.8	9.1	184.5	10.1	188.3	8.2	185.5	10.5	0.889	0.395	0.08
CMJ (Δ cm)	−10.2	8.8	−6.8	4.8	−13.3	8.7	−6.8	5.9	4.564	0.008	0.28
Lactate (mmoL/L)	19.1	4.7	19.0	4.2	20.1	4.6	17.8	4.4	0.979	0.413	0.07

RPE: Rate of perceived exertion; CMJ: countermovement jump.

**Table 2:** Sleep quality results.

	Placebo		Caffeine			Effect Size (ES)	95% CI for ES	
	Mean	SD	Mean	SD	*p*		Lower	Upper
**Actigraphic sleep quality**								
Latency (min)	6.15	2.79	6.77	2.32	0.290	−0.31	−0.86	0.25
Sleep efficiency (%)	92.2	3.0	86.4	5.5	0.003	0.71	0.27	0.91
Time in bed (min)	470.2	118.3	461.2	128.2	0.641	0.13	−0.42	0.68
Actual sleep time (min)	434.8	119.7	402.3	136.3	0.091	0.51	−0.08	1.08
Actual wake time (min)	29.2	15.4	52.1	23.2	0.001	−1.18	−1.89	−0.45
Awakenings (n)	13.62	7.05	18.85	7.50	0.005	−0.96	−1.61	−0.28
Average time of each awakening (min)	2.79	1.90	3.18	1.72	0.402	−0.24	−0.79	0.32
**Karolinska Sleep Questionnaire**								
Sleep quality	3.36	0.75	2.21	0.98	0.005	1.11	0.43	1.77
Calm sleep	3.50	1.09	2.36	1.15	0.005	1.11	0.43	1.77
Ease of falling asleep	3.43	1.22	1.57	0.85	0.003	1.38	0.62	2.10
Amount of dreaming	1.43	0.76	1.07	0.48	0.120	0.48	−0.08	1.03
Ease of waking up	3.43	0.76	3.14	0.86	0.395	0.24	−0.30	0.76
Feeling refreshed after awakening	2.07	0.73	1.50	0.65	0.006	1.11	0.43	1.77
Slept throughout the time allotted	3.14	0.86	2.79	1.89	0.389	0.24	−0.30	0.77

**Table 3:** Heart rate variability results during the night after placebo or caffeine ingestion.

	Placebo		Caffeine			Effect Size (ES)	95% CI for ES	
	Mean	SD	Mean	SD	*p*		Lower	Upper
Mean R-R (ms)	1151.5	114.4	1184.7	131.1	1.000	−0.21	−0.80	0.39
SDNN (ms)	40.4	7.2	36.5	6.7	0.102	0.56	−0.58	0.58
HR (bpm)	52.4	5.7	51.2	6.1	1.000	0.58	−0.01	0.87
RMSSD (ms)	27.1	4.1	26.9	4.0	0.715	0.11	−0.48	0.70
pNN50 (%)	7.1	3.3	7.2	3.1	0.956	−0.02	−0.61	0.58
LF (ms^2^)	986.3	617.3	814.0	377.4	0.205	0.41	−0.22	1.02
HF (ms^2^)	192.4	111.1	190.7	77.8	0.953	0.02	−0.57	0.61
TP (ms)	1689.1	1094.3	1371.7	639.8	0.214	0.40	−0.23	1.08
LF/HF	5.6	2.5	4.6	2.0	0.182	0.43	−0.20	1.04

SD: standard deviation; SDNN: standard deviation of all normal N–N intervals; HR: mean heart rate; RMSSD: square root of the mean of the sum of the squared differences between adjacent normal R–R intervals; pNN50: percentage of differences between adjacent normal R–R intervals > 50 ms; TP: Total power; LF: low frequency; HF: high frequency (HF).
